# Psychosocial Determinants of Vaccine Hesitancy and Vaccination Behavior Among Older Adults in China: A Large Cross-Sectional Study

**DOI:** 10.3389/ijph.2026.1608705

**Published:** 2026-02-16

**Authors:** Rui Peng, Zongchao Peng, Siwen Huang, Sitong Luo, Dong Guo

**Affiliations:** 1 School of Government and Public Affairs, Communication University of China, Beijing, China; 2 Center for Crisis Management Research, Tsinghua University, Beijing, China; 3 School of Public Policy and Management, Tsinghua University, Beijing, China; 4 Vanke School of Public Health, Tsinghua University, Beijing, China; 5 Institute for Healthy China, Tsinghua University, Beijing, China; 6 School of Economics and Management, Communication University of China, Beijing, China

**Keywords:** vaccine hesitancy, vaccination behavior, older adults, health belief model, theory of planned behavior

## Abstract

**Objectives:**

To identify psychosocial determinants of vaccine hesitancy and vaccination behavior among older adults in China, using an integrated framework of the Health Belief Model and Theory of Planned Behavior.

**Methods:**

We conducted a cross-sectional survey targeting individuals aged 60+ years during the pandemic vaccine rollout. The analysis included Probit regression models based on the Health Belief Model (HBM), Theory of Planned Behaviour (TPB), risk perception, vaccine confidence and behavioral intervention, with demographic and health status as control variables.

**Results:**

Among older adults, vaccine hesitancy was significantly associated with perceived benefits, perceived barriers, attitude, self-efficacy, concerns about vaccine safety, perceived vaccine necessity, positive incentives, negative social pressure, information prompts, and vaccination reminders. Actual vaccination behavior was significantly influenced by vaccine hesitancy, perceived severity, perceived benefits, perceived barriers, attitude, self-efficacy, and positive incentives, etc. Age and medical contraindications significantly affected both hesitancy and vaccine behavior.

**Conclusion:**

The integrated theoretical framework reveals age-specific behavioral pathways that are critical to vaccine acceptance among older Chinese adults. These findings underscore the importance of age-tailored interventions that address psychosocial barriers and leverage timely behavioral nudges to improve immunization outcomes in aging populations.

## Introduction

Since the World Health Organization (WHO) announced the global pandemic in response to the rapid international spread of a novel coronavirus [1], this public health crisis has been widely associated with severe disruptions to health systems and potentially catastrophic consequences for population health and wellbeing [2]. Since the start of the pandemic, age has been outlined as the critical determinant in the pandemic patients, and statistical data have suggested that older people, defined as those aged 60 years or above, are the most vulnerable group in the infected population [3–5]. Infection with the pandemic in older adults, especially males, has been associated with more severe symptoms and higher mortality risk [6–9]. People aged 65 years and over accounted for about 80% of all the pandemic deaths in the United States, and people aged over 85 years had a 630 times mortality rate compared with those aged 18–29 years [10]. Most deaths have been reported among people aged 70 years and above in Australia.

The late 18th century witnessed the emergence of safe vaccinations. Since then, investing in them has been of vital importance in combating infectious diseases like polio and smallpox. Moreover, vaccination is regarded as the optimal and cost-effective approach to achieving herd immunity [11]. The rates of vaccine coverage serve as the decisive factor in effectively controlling the pandemic.

Despite the rapid development and widespread availability of vaccines against the novel coronavirus, vaccine hesitancy has persisted as a significant public health challenge. It hinders efforts to achieve high vaccination coverage against highly transmissible diseases and has emerged as a global public health concern in the context of the pandemic [12]. According to the World Health Organization’s Strategic Advisory Group of Experts (SAGE), vaccine hesitancy is defined as the delay in acceptance or refusal of vaccines despite the availability of vaccination services [13]. Moreover, acceptance rates for the pandemic vaccines continue to vary substantially across age groups [14–17]. Among older adults, pronounced vaccine hesitancy and uncertainty have been linked to disproportionately high mortality rates—indeed, some regions with low uptake in this demographic have experienced the world’s highest *per capita* daily death tolls. Therefore, understanding and addressing vaccine hesitancy, particularly among older individuals, is imperative for the success of global immunization strategies [18].

The integration of the Health Belief Model (HBM) and the Theory of Planned Behavior (TPB) is theoretically justified by their complementary explanatory strengths in understanding vaccination behavior [19–23]. The HBM is one of the most widely used models in vaccination behavior research, posits that health actions are driven by perceived susceptibility to disease, perceived benefits and barriers of vaccination, and cues to action [24]. This model has been extensively validated in predicting vaccination intentions and behaviors across diverse contexts, including influenza, swine flu, hepatitis B, and the pandemic [25–28]. Meanwhile, the TPB asserts that behavioral intention is determined by attitude, subjective norms, and perceived behavioral control, with actual behavior being influenced by intention [29, 30]. The model emphasizes a positive correlation between intention and behavior, where enhanced intention facilitates more proactive health behaviors [29, 30]. While both models are frequently employed in public health research to examine health behaviors [31, 32], they address distinct dimensions: the HBM focuses on risk perception and benefit-risk evaluation, whereas the TPB emphasizes the intention-behavior pathway. Empirical evidence indicates that vaccination behavior cannot be fully captured by either model alone, as HBM accounts for threat appraisal while TPB explains behavioral enactment. Therefore, integrating these frameworks provides a more comprehensive analytical lens for examining the full continuum of vaccine decision-making, from risk assessment to behavioral action, thereby strengthening the theoretical foundation for intervention design.

Perceived risk of the pandemic and vaccine confidence are key factors influencing vaccination intention and behavior. Risk perception refers to individuals’ subjective judgments about the severity and likelihood of a health threat, and studies confirm that greater concern about disease severity promotes preventive actions, including vaccination [33]. Vaccine confidence encompasses beliefs, emotions, and attitudes toward vaccines, particularly trust in their importance, safety, efficacy, and compatibility with personal or religious values [34]. Behavioral interventions are also critical in promoting health behaviors. Within the Health Belief Model (HBM), cues to action—such as vaccination reminders, informational posters, public campaigns, and incentive systems—have been shown to significantly increase vaccine uptake by enhancing threat awareness and prompting behavioral response [35, 36]. In summary, it is essential to incorporate both pandemic risk perception and vaccine confidence into analytical models. Moreover, cues to action should be further differentiated to reflect real-world interventions. This study integrates commonly used behavioral prompts in China’s vaccination campaign—alongside constructs from the HBM and Theory of Planned Behavior (TPB)—into a comprehensive framework to examine the determinants of the pandemic vaccination intention and behavior.

While the literature regarding the pandemic vaccine hesitancy and vaccination behaviour has dramatically increased, most of the studies have focused on young adults rather than the elderly [37–40]. Although few existing studies have investigated the attributes and barriers associated with vaccinations for the pandemic, seasonal influenza and other vaccinations in older adults globally [16, 41–43], due to the difficulty in collecting samples [44, 45], the more specific characteristics of vaccine hesitancy and vaccination behaviour of older adults in different age groups, as well as the differences in influencing factors between older adults and other populations are still poorly understood.

Building on existing literature, this study focuses on older Chinese adults to examine the determinants of the pandemic vaccine hesitancy and vaccination behavior, particularly in relation to psychological characteristics, social influences, and behavioral interventions. A cross-sectional survey was conducted during the period of China’s nationwide vaccination campaign targeting adults aged 60 and older, which coincided with the concurrent rollout of booster doses for individuals aged 18–60. Participants were questioned regarding their the pandemic vaccination willingness and current vaccination status. To analyze the variables influencing these factors, probit regression was employed, incorporating aspects such as risk perception, vaccine-related beliefs, the Health Belief Model (HBM), the Theory of Planned Behaviour (TPB), and behavioral interventions. The regression model also accounted for demographic variables and physical health conditions as control factors. Given that older adults are becoming an increasingly challenging demographic to reach in vaccination campaigns, comprehending their attitudes toward the pandemic vaccines and identifying the obstacles to vaccination is of paramount importance. This study focused on determining the predictors of the pandemic vaccination willingness and behavior in the elderly population and exploring how these factors differ from those observed in younger adults within China.

## Methods

Ethical approval was obtained from the Institutional Review Board of Tsinghua University, China (Approval Number: 20250008).

### Study Design and Data Collection

This cross-sectional study examined factors influencing vaccine hesitancy and vaccination behavior among adults in mainland China. The survey covered 28 provinces, municipalities, and regions across the country, with the majority of responses collected from C city. Data were collected during the phase of China’s national immunization campaign that prioritized booster doses for adults aged 18–60 years and both primary and booster vaccination for those aged ≥60 years. Participants were recruited via stratified random sampling through Wen JuanXing (WJX), with age-stratified quotas designed to reflect the operational distribution of the campaign. A total of 8,088 questionnaires were initially collected. After excluding invalid responses (based on duplicate IP addresses, completion times <150 s, and respondents <18 years), 6,525 valid questionnaires (80.7%) were retained for analysis.

The final sample comprised 4,189 participants aged 18–60 years (64.2%) and 2,336 aged ≥60 years (35.8%). This age distribution aligns with real-world vaccination coverage proportions observed in C city’s operational context: in L Street (a representative district), the ratio of 18–60-year-olds receiving booster doses (46,536 individuals) to 60-year-olds receiving primary/booster vaccinations (24,637 individuals) was 65.4% and 34.6%. The 35.8% representation of ≥60-year-olds in our sample closely mirrors this district-level data (34.6%), confirming the sample’s alignment with the campaign’s target population distribution. Crucially, inclusion of the 18–60-year-old cohort enabled comparative analysis of vaccination determinants across age groups, allowing for the identification of age-specific behavioral patterns in Health Belief Model (HBM) and Theory of Planned Behavior (TPB) constructs that would be obscured in a single-age-group study.

### Survey Measures

For measurement, we adapted established scales from prior studies, modifying them to reflect the context of the pandemic. The final questionnaire was developed through expert consultation and iterative pre-testing.

This study included two dependent variables: Vaccine Hesitancy and Vaccination Behavior. Vaccine Hesitancy was assessed using two 5-point Likert-scale items [46–48]: “To what extent are you willing to be vaccinated against the pandemic?” and “To what extent do you think your family members are willing to be vaccinated against the pandemic?” Response options were: absolutely not willing, may be unwilling, not sure, may be willing, and very willing. Following existing literature, which commonly evaluates vaccination willingness among close contacts such as family members, partners, or children, we constructed a composite hesitancy score based on respondents’ self-reported willingness and their perception of family members’ willingness using factor analysis. Higher scores indicate lower vaccine hesitancy. The scale showed acceptable internal consistency (Cronbach’s alpha = 0.729). Vaccination Behavior was assessed with the question [49–51]: “What is your current vaccination status?” Response options were: no vaccine administered, first dose administered, two doses administered, and booster dose administered.

The independent variables of this study encompassed psychological characteristics (variables related to the HBM and the TPB model), Risk Perception, Vaccine Confidence, and Behavioral Interventions. These variables were chosen in part based on existing literature on vaccine hesitancy and vaccination behaviors [52], with a focus on perceptions of the pandemic and vaccines, psychological characteristics, and policy interventions.

Regarding the HBM-related variables, four factors were considered to determine an individual’s decision to adopt a health-promoting behavior [24, 53–55]: (1) the Perceived Susceptibility of the adverse health condition being avoided; (2) the Perceived Severity to the adverse condition; (3) the Perceived Benefits the person associates with the behavior; and (4) the Perceived Barriers to adopting the protective behavior. We measured agreement with four statements we designed: “I am in a high-risk group, and I am afraid of being infected with the virus”; “I am afraid of the consequences if I do not get the vaccine and am infected with the virus”; “I want to be able to go out conveniently”; “I am concerned about the side effects of vaccination.” Although the TPB informed our variable selection, we did not include all its constructs. The specific measurement of cues to action variables will be elaborated in the following Behavioral Interventions section.

For the TPB-related variables, a model was used in which cognitions and broader constructs (i.e., Attitude, Perceived Behavioral Control, and Self-Efficacy) influence behavior. We designed four statements to measure these constructs [30, 56, 57]: “vaccination is time-consuming”; “the decision to vaccinate or not is entirely up to me”; “I do not need to be vaccinated if I take the necessary precautions”; “as a citizen, it is my vaccination is a matter of no concern to me.”

To assess Risk Perception, we used two dimensions: pandemic concern and pandemic severity, with the questions [58, 59]: “I often follow the latest news of the pandemic at home and abroad” and “I think the current pandemic at home and abroad is severe.” Vaccine Confidence was measured by agreement with four statements [60]: “the Chinese vaccine is safe, and there is no risk to my health” (vaccine safety); “vaccination is effective in preventing infection or severe symptoms” (vaccine effectiveness); and “vaccination is necessary to increase protection” (vaccine necessity.

Finally, we examined the impact of Behavioral Interventions from four perspectives [61–63]: Positive Incentives, represented by the statement “get small gifts and other rewards”; Negative Pressure, represented by the statement “I was required to be vaccinated”; Information Prompts, including posters, banners, electronic screens, WeChat official accounts, WeChat circles, web news, and television news (Cronbach alpha = 0.862); and Vaccination Prompts, including WeChat group reminders, message reminders, telephone reminders, posters at the individual’s door, a reminder by the staff of the individual’s residence, a reminder by the staff at the individual’s place of work, a reminder by family members, and a reminder by friends (Cronbach alpha = 0.898). We assessed all independent variables on a 5-point scale ranging from *very low* to *very high*.

The control variables in our study were participants’ demographic characteristics and any contraindications to vaccination. Demographic characteristics included gender, age, educational level, political status, and income. We also asked participants whether they had not received the vaccine because of contraindications.

### Statistical Analyses

Descriptive statistics were used to summarize demographic characteristics and survey responses. Chi-square tests were applied for categorical variables and ANOVA for continuous variables to assess differences across groups. Statistical significance was defined at a two-tailed p < 0.1. Factor analysis was conducted to evaluate the reliability and validity of Likert-scale items, beginning with an assessment of inter-item correlations followed by exploratory factor analysis. A Probit regression model was employed to examine the influence of control variables on vaccine hesitancy and vaccination behavior. This model estimated the effects of key independent variables—including constructs from the Health Belief Model (HBM) and Theory of Planned Behavior (TPB), risk perception, vaccine confidence, and behavioral interventions—while adjusting for gender, age, education, political status, income, and medical contraindications. All analyses were performed using Stata MP 17.0.

For reliability assessment, Cronbach’s alpha was used to evaluate internal consistency of the Likert-scale constructs, as recommended for psychometric scales in survey research [64]. The Cronbach’s alpha values ranged from 0.729 to 0.898 (see Table 1). As values above 0.7 indicate acceptable internal consistency, all scales demonstrated good reliability.

**TABLE 1:** Reliability test results of subjective perception variables (Beijing, China, 2022).

Variables		Items	Cronbach’s alpha
Dependent variable	Vaccine hesitancy	2	0.729
Independent variables	Self-efficacy	2	0.847
	Vaccine cognition	3	0.884
	Information prompt	3	0.862
	Vaccination prompt	8	0.898

Survey data were collected in C city, China, during the national immunization campaign that prioritized primary and booster vaccination for adults aged ≥60 years and booster doses for adults aged 18–60 years.

For validity, the Kaiser-Meyer-Olkin (KMO) measure of sampling adequacy and Bartlett’s test of sphericity were used to assess suitability for factor analysis. All scales met the KMO criterion (>0.6), and Bartlett’s tests were statistically significant (p < 0.001), supporting the factorability of the data. To address common method bias, both procedural and statistical controls were implemented. Procedurally, the survey was administered anonymously to reduce response bias; reverse-coded items were included to minimize acquiescence bias; variable labels were omitted and item order randomized to conceal the study’s focus and enhance response authenticity. Statistically, multicollinearity was assessed using variance inflation factor (VIF) and tolerance (TOL). All TOL values exceeded 0.1 and VIF values remained below 3.08 (mean VIF = 1.76), indicating no significant multicollinearity. Harman’s single-factor test was also conducted. The first unrotated principal component explained 24.2% of the total variance (eigenvalue = 2.909), which is below the 40% threshold, suggesting no substantial common method variance.

## Results

### Descriptive Statistics of Vaccine Hesitancy and Vaccination Behaviour

Among the 6,532 valid questionnaires, 6,059 (92.65%) respondents indicated they would be “very willing” to be vaccinated against the pandemic, while 4.95% stated they “may be willing to be vaccinated.” Regarding family vaccination willingness, 5,704 (87.23%) respondents reported that their families were “very willing” to be vaccinated, and 9.74% thought their families “might be willing” to get vaccinated. In terms of vaccination status, 5,364 (82.02%) participants had received a booster dose of the vaccine, 852 (13.03%) had received two doses, and 101 (1.54%) had received one dose. Only 3.41% of participants remained unvaccinated. The number of respondents in different age groups was as follows: 4,204 (64.28%) aged 18–60 years, 879 (13.44%) aged 61–65 years, 1,266 (19.36%) aged 66–75 years, and 191 (2.92%) aged 75 years and older.

Table 2 provides an overview of the frequencies and percentages of reported hesitancy to get vaccinated against the pandemic and vaccination behavior, categorized by age. The data indicate a high willingness to be vaccinated and active vaccination behavior among respondents, consistent with the high vaccination coverage rates for C city reported by the China CDC during the national rollout of primary and booster doses for older adults.

**TABLE 2:** Demographic characteristics of respondents (n = 8,525) (Beijing, China, 2022).

Characteristic	<60	>60				Pearson Chi [2]	P-value	Study cohort
		Total	60–65	65–75	>75			
To what extent are you willing to be vaccinated against the pandemic?								
Absolutely not willing to vaccinate	12 (75.00)	4 (25.00)	2 (12.50)	2 (12.50)	0 (0.00)	16.04	0.0030	16
May be unwilling to vaccinate	25 (80.65)	6 (19.35)	2 (6.45)	4 (12.90)	0 (0.00)			31
Not sure	81 (73.64)	29 (26.36)	15 (13.64)	14 (12.73)	0 (0.00)			110
May be willing to vaccinate	230 (70.99)	94 (29.01)	35 (10.80)	50 (15.43)	9 (2.78)			324
Very willing to vaccinate	3,856 (63.64)	2,203 (36.36)	825 (13.62)	1,196 (19.74)	182 (3.00)			6,059
To what extent do you think your family is willing to be vaccinated against the pandemic?								
Absolutely not willing to vaccinate	8 (57.14)	6 (42.86)	2 (14.29)	3 (21.43)	1 (7.14)	9.75	0.0448	14
May be unwilling to vaccinate	27 (77.14)	8 (22.86)	4 (11.43)	4 (11.43)	0 (0.00)			35
Not sure	105 (70.47)	44 (29.53)	16 (10.74)	23 (15.44)	5 (3.36)			149
May be willing to vaccinate	432 (67.82)	205 (32.18)	88 (13.81)	102 (16.01)	15 (2.35)			637
Very willing to vaccinate	3,631 (63.66)	2073 (36.34)	769 (13.48)	1,134 (19.88)	170 (2.98)			5,704
What is your current pandemic vaccination status?								
No vaccination has been administered	58 (57.43)	43 (42.57)	15 (14.85)	20 (19.80)	8 (7.92)	25.22	<0.0001	101
The first dose of the vaccine has been administered	512 (60.09)	340 (39.91)	124 (14.55)	185 (21.71)	31 (3.64)			852
Two doses of the vaccine have been administered	3,522 (65.66)	1842 (34.34)	687 (12.81)	1,014 (18.90)	141 (2.63)			5,364
A booster dose of the vaccine has been administered	112 (50.22)	111 (49.78)	53 (23.77)	47 (21.08)	11 (4.93)			223

Data collected in C city, China, during the national elderly and booster vaccination campaign (see Table 1 for details).

Pearson Chi-square tests and P-value tests apply to continuous variables in groups of ages 18–60 and over 60 years.

First column has *frequencies* and second column in parentheses has *percentages* (%).

### Analyses of the Effects of HBM, TPB, and Other Factors on Vaccine Hesitancy and Vaccination Behavior Among Older Adults

As previously noted, following assessments of reliability, validity, and potential common method bias, this study examines the associations among variables using correlation analysis and multivariate Probit regression. To evaluate the statistical associations of constructs from the Health Belief Model (HBM), the Theory of Planned Behavior (TPB), and risk perception with vaccine hesitancy, we control for demographic characteristics and the presence of contraindications. Table 3 presents separate Probit regression results for vaccine hesitancy and vaccination behavior, with each outcome estimated across three progressively specified models (Model 1 to Model 3) and stratified by four age groups: the full adult sample (age ≥18) and three subgroups of older adults (61–65, 66–75, and over 75 years).

**TABLE 3:** Probit regression analysis to predict vaccine hesitancy and vaccination behavior among older adults (n = 8,525) (Beijing, China, 2022).

Variables	Vaccine hesitancy				Vaccination behaviour			
	(1)	(2)	(3)	(4)	(1)	(2)	(3)	(4)
	≥18	61–65	66–75	>75	≥18	61–65	66–75	>75
Vaccine hesitancy	​	​	​	​	0.243*** (0.016)	0.248*** (0.043)	0.371*** (0.044)	0.241* (0.129)
Model 1: Health belief model constructs								
Perceived susceptibility	0.049*** (0.016)	0.090** (0.043)	0.041 (0.037)	0.007 (0.094)	0.007 (0.015)	−0.080** (0.039)	−0.002 (0.032)	−0.009 (0.074)
Perceived severity	0.045*** (0.015)	0.067* (0.039)	0.051 (0.034)	0.043 (0.097)	0.065*** (0.014)	0.091** (0.036)	0.035 (0.029)	0.006 (0.073)
Perceived benefits	−0.019 (0.014)	−0.104*** (0.038)	−0.014 (0.032)	0.015 (0.086)	−0.025* (0.013)	0.006 (0.034)	−0.020 (0.028)	0.016 (0.070)
Perceived barriers	−0.243*** (0.018)	−0.109** (0.047)	−0.199*** (0.043)	−0.206* (0.105)	0.052*** (0.017)	0.042 (0.042)	0.040 (0.038)	−0.084 (0.084)
Model 2: Theory of planned behavior constructs								
Attitude	−0.018 (0.020)	0.071 (0.052)	−0.015 (0.045)	−0.266** (0.111)	0.104*** (0.019)	0.162*** (0.049)	0.060 (0.040)	0.067 (0.093)
Perceived behaviour control	0.033** (0.014)	−0.016 (0.040)	0.009 (0.035)	−0.079 (0.101)	0.021 (0.013)	0.090** (0.035)	0.028 (0.029)	0.036 (0.069)
Self-efficacy	−0.253*** (0.025)	−0.143** (0.068)	−0.201*** (0.054)	−0.048 (0.144)	0.083*** (0.025)	−0.096 (0.063)	0.069 (0.052)	−0.012 (0.115)
Model 3: Risk Perception + Vaccine Cognition + Behavioural interventions								
Pandemic concern	0.130*** (0.031)	0.130 (0.119)	−0.038 (0.118)	0.100 (0.349)	−0.001 (0.033)	−0.022 (0.117)	−0.051 (0.117)	0.178 (0.293)
Pandemic severity	0.054*** (0.019)	0.118** (0.050)	0.036 (0.048)	0.110 (0.166)	0.029 (0.018)	0.080* (0.047)	0.119*** (0.039)	−0.222 (0.157)
Vaccine safety	0.151*** (0.038)	0.096 (0.099)	0.185* (0.096)	−0.198 (0.399)	0.031 (0.038)	0.076 (0.091)	0.042 (0.095)	0.384 (0.281)
Vaccine effectiveness	0.049 (0.038)	0.127 (0.102)	0.116 (0.084)	0.088 (0.212)	−0.026 (0.039)	0.106 (0.095)	0.025 (0.081)	−0.020 (0.190)
Vaccine necessity	0.372*** (0.045)	0.371*** (0.132)	0.321*** (0.113)	1.034*** (0.380)	−0.030 (0.049)	−0.152 (0.133)	−0.101 (0.118)	−0.324 (0.385)
Positive incentives	−0.002 (0.018)	−0.062 (0.050)	−0.081* (0.046)	0.209 (0.183)	0.003 (0.016)	−0.056 (0.043)	−0.017 (0.038)	0.054 (0.116)
Negative pressure	−0.039*** (0.014)	−0.039 (0.038)	0.047 (0.038)	−0.172 (0.109)	0.025** (0.013)	−0.008 (0.033)	−0.005 (0.030)	−0.000 (0.082)
Information prompt	0.059** (0.023)	−0.041 (0.064)	0.129** (0.054)	0.105 (0.148)	−0.029 (0.022)	−0.020 (0.059)	−0.030 (0.049)	0.000 (0.103)
Vaccination prompt	0.181*** (0.025)	0.317*** (0.068)	0.144** (0.062)	0.278 (0.171)	0.089*** (0.023)	0.082 (0.061)	0.092* (0.054)	−0.219* (0.114)
All controls	Yes	Yes	Yes	Yes	Yes	Yes	Yes	Yes
Observations	6,525	879	1,266	191	6,525	879	1,266	191
Pseudo R-squared	Model 1:0.051 Model 2:0.044 Model 3:0.114	Model 1:0.043 Model 2:0.030 Model 3:0.110	Model 1:0.077 Model 2:0.051 Model 3:0.106	Model 1:0.034 Model 2:0.053 Model 3:0.144	Model 1:0.102 Model 2:0.108 Model 3:0.100	Model 1:0.099 Model 2:0.110 Model 3:0.099	Model 1:0.111 Model 2:0.114 Model 3:0.117	Model 1:0.063 Model 2:0.063 Model 3:0.091

Note. Data collected in C city, China, during the national elderly and booster vaccination campaign (see Table 1 for details).

Standard errors in parentheses *p < 0.1, **p < 0.05, ***p < 0.01.

We first examine the factors associated with vaccine hesitancy across three model specifications and age groups. In Model 1 (HBM), the set of explanatory variables is associated with vaccine hesitancy in the full sample, with a Pseudo R-squared of 0.051. Within this model, Perceived Susceptibility (β = 0.049, p < 0.01) and Perceived Barriers (β = −0.243, p < 0.01) are statistically significantly associated with higher levels of vaccine hesitancy. Model 2 (TPB) yields a Pseudo R-squared of 0.044. Perceived Behavioral Control (β = 0.033, p < 0.05) and Self-Efficacy (β = −0.253, p < 0.01) are significantly associated with vaccine hesitancy in the full sample. Model 3 (Risk Perception, Vaccine Cognition, and Behavioral Interventions) demonstrates the highest fit, with a Pseudo R-squared of 0.114. Most variables in this model show statistically significant associations with vaccine hesitancy. Specifically, Pandemic Concern (β = 0.130), Pandemic Severity (β = 0.054), Vaccine Safety (β = 0.151), Vaccine Necessity (β = 0.372), and Vaccination Prompt (β = 0.181) are positively associated with vaccine hesitancy (p < 0.01), while Negative Peer Pressure (β = −0.039, p < 0.01) is negatively associated. Notably, Vaccine Necessity is consistently significant across all older adult age groups and exhibits the largest coefficient among individuals aged 75 and older (β = 1.034, p < 0.01). Additionally, Perceived Severity, Vaccine Safety, Positive Incentives, Information Prompt, and Vaccination Prompt are associated with vaccine hesitancy in specific older adult subgroups.

We next present the correlates of actual vaccination behavior using the same modeling approach. Vaccine hesitancy is marginally associated with lower vaccination uptake in the full sample and across older adult age groups (p < 0.10), suggesting a directional relationship between intention and behavior. In the HBM model, the explanatory variables are associated with vaccination behavior, with a Pseudo R-squared of 0.102. Perceived Severity is positively and significantly associated with vaccination in the full sample (β = 0.067, p < 0.01). Among older adults, Perceived Susceptibility, Perceived Severity, and Perceived Benefits show associations with vaccination behavior. The TPB model explains 10.8% of the variation (Pseudo R-squared = 0.108). Attitude (β = 0.104, p < 0.01) is significantly associated with vaccination in the full sample. Other TPB constructs show associations in younger elderly subgroups (under 75), but none of the TPB variables are statistically significant among adults aged 75 and older. In Model 3, the set of variables is associated with vaccination behavior, with a Pseudo R-squared of 0.100. Vaccine Safety, Vaccine Necessity, Negative Peer Pressure, and Vaccination Prompt are marginally associated with vaccination (p < 0.10). Overall, the strength of the associations, as indicated by the Pseudo R-squared values, is greater in the full sample than in the stratified older adult groups.

### Analyses and Comparison of the Effects of Socio-Ecological Factors on Vaccine Hesitancy and Vaccination Behavior Among Older Adults

To provide a clearer depiction of the impact of all variables on vaccine hesitancy and vaccination behavior among older adults, Table 4 presents the results from Probit regression analysis incorporating all variables into the model. To quantify the actual effects of these variables, we further report the average marginal effects (AME) in Tables 5, 6 for vaccine hesitancy and vaccination behavior, respectively. To analyze the intrinsic characteristics of different age groups within the older adult population, we stratify the sample into three age groups: 61–65, 66–75, and over 75 years. The full sample (age ≥18) is also included to facilitate comparisons across age groups.

**TABLE 4:** Probit regression analysis and comparison of all variables predicting vaccine hesitancy and vaccination behavior among older adults (n = 8,525) (Beijing, China, 2022).

Vaccine hesitancy					Vaccination behaviour			
Variables	(1)	(2)	(3)	(4)	(1)	(2)	(3)	(4)
	≥18	61–65	66–75	>75	≥18	61–65	66–75	>75
Vaccine hesitancy	​				0.244*** (0.018)	0.221*** (0.048)	0.379*** (0.047)	0.411*** (0.148)
Health belief model (HBM) related variables								
Perceived susceptibility	0.023 (0.017)	0.073 (0.047)	0.027 (0.041)	−0.146 (0.122)	0.001 (0.015)	−0.061 (0.041)	0.002 (0.033)	0.013 (0.081)
Perceived severity	0.017 (0.017)	0.052 (0.045)	−0.008 (0.038)	0.125 (0.127)	0.063*** (0.015)	0.103*** (0.039)	0.027 (0.030)	0.022 (0.080)
Perceived benefits	−0.021 (0.017)	−0.072 (0.047)	−0.065* (0.039)	0.174 (0.135)	−0.036** (0.016)	0.019 (0.042)	−0.020 (0.033)	−0.013 (0.085)
Perceived barriers	−0.155*** (0.023)	−0.106* (0.064)	−0.139** (0.055)	−0.074 (0.173)	−0.054** (0.021)	−0.095* (0.057)	−0.032 (0.046)	−0.212* (0.124)
Theory of planned behaviour (TPB) related variables								
Attitude	0.045* (0.023)	0.140** (0.066)	0.002 (0.053)	−0.447** (0.176)	0.123*** (0.022)	0.220*** (0.059)	0.070 (0.045)	0.226* (0.120)
Perceived behaviour control	0.021 (0.016)	−0.025 (0.046)	−0.005 (0.040)	−0.203 (0.135)	0.029** (0.013)	0.096*** (0.037)	0.026 (0.030)	0.042 (0.074)
Self-efficacy	−0.183*** (0.028)	−0.076 (0.077)	−0.130** (0.062)	−0.063 (0.189)	0.103*** (0.026)	−0.046 (0.067)	0.090 (0.056)	0.072 (0.138)
Risk perception								
Pandemic concern	0.105*** (0.031)	0.132 (0.121)	−0.049 (0.122)	0.360 (0.386)	0.012 (0.033)	−0.003 (0.122)	−0.014 (0.119)	0.194 (0.304)
Pandemic severity	0.059*** (0.019)	0.107** (0.052)	0.043 (0.050)	0.132 (0.187)	0.020 (0.018)	0.071 (0.048)	0.111*** (0.039)	−0.228 (0.166)
Vaccine cognition								
Vaccine safety	0.126*** (0.038)	0.092 (0.099)	0.172* (0.098)	−0.723 (0.473)	0.013 (0.039)	0.102 (0.092)	0.031 (0.096)	0.398 (0.292)
Vaccine effectiveness	0.058 (0.039)	0.134 (0.104)	0.110 (0.085)	0.262 (0.240)	−0.023 (0.039)	0.111 (0.097)	0.023 (0.082)	−0.055 (0.201)
Vaccine necessity	0.341*** (0.046)	0.348*** (0.134)	0.302*** (0.115)	1.597*** (0.446)	−0.009 (0.049)	−0.183 (0.136)	−0.079 (0.119)	−0.416 (0.391)
Behavioural interventions								
Positive incentives	0.029 (0.020)	−0.098* (0.056)	−0.040 (0.049)	0.416* (0.234)	−0.051*** (0.018)	−0.109** (0.047)	−0.054 (0.040)	0.044 (0.122)
Negative pressure	−0.018 (0.019)	−0.025 (0.051)	0.099** (0.048)	−0.237* (0.143)	0.023 (0.017)	−0.029 (0.046)	−0.006 (0.038)	−0.017 (0.097)
Information prompt	0.062*** (0.024)	−0.030 (0.065)	0.118** (0.055)	−0.001 (0.180)	−0.022 (0.023)	−0.021 (0.060)	−0.021 (0.050)	−0.005 (0.113)
Vaccination prompt	0.173*** (0.026)	0.288*** (0.069)	0.171*** (0.063)	0.253 (0.191)	0.070*** (0.023)	0.066 (0.062)	0.072 (0.054)	−0.188 (0.119)
Demographic characteristic								
Gender	0.018 (0.046)	−0.142 (0.141)	0.064 (0.115)	−0.724** (0.338)	0.244*** (0.018)	−0.147 (0.124)	−0.142 (0.100)	0.095 (0.237)
Age	0.001 (0.002)	−0.026 (0.039)	−0.047** (0.019)	0.028 (0.047)	−0.142*** (0.043)	−0.059* (0.035)	−0.016 (0.016)	−0.077** (0.032)
Education	−0.025 (0.023)	0.044 (0.066)	0.044 (0.055)	−0.128 (0.165)	−0.004*** (0.002)	−0.033 (0.058)	−0.017 (0.045)	0.010 (0.108)
Political statue	0.001 (0.015)	0.092** (0.042)	0.004 (0.038)	0.361* (0.188)	−0.022 (0.020)	−0.023 (0.036)	−0.029 (0.032)	−0.094 (0.088)
Income	0.017 (0.015)	0.007 (0.054)	0.038 (0.056)	−0.098 (0.144)	−0.030** (0.014)	−0.023 (0.048)	0.003 (0.044)	0.103 (0.105)
Contraindication	−0.107*** (0.019)	−0.205*** (0.048)	−0.142*** (0.043)	0.154 (0.146)	0.016 (0.014)	−0.312*** (0.043)	−0.287*** (0.036)	−0.261*** (0.089)
Observations	6,525	879	1,266	191	6,525	879	1,266	191
Pseudo R-squared	0.130	0.114	0.123	0.107	0.130	0.114	0.123	0.107

Data collected in C city, China, during the national elderly and booster vaccination campaign (see Table 1 for details).

Standard errors in parentheses *p < 0.1, **p < 0.05, ***p < 0.01.

**TABLE 5:** Average marginal effects from the probit model of vaccine hesitancy among older adults (n = 8,525). (Beijing, China, 2022).

Vaccine hesitancy								
Variables	≥18		61–65		66–75		>75	
	AME	95% CI	AME	95% CI	AME	95% CI	AME	95% CI
Health belief model (HBM) related variables								
Perceived susceptibility	0.021**	[0.005, 0.037]	0.0442**	[0.006, 0.083]	0.012	[−0.020, 0.043]	−0.007	[−0.071, 0.057]
Perceived severity	0.002	[−0.013, 0.017]	−0.00337	[−0.039, 0.032]	−0.008	[−0.035, 0.019]	0.014	[−0.042, 0.070]
Perceived benefits	−0.017*	[−0.035, 0.001]	−0.0412*	[−0.089, 0.007]	−0.037**	[−0.067, −0.006]	0.043	[−0.030, 0.116]
Perceived barriers	−0.068***	[−0.096, −0.040]	−0.0539	[−0.139, 0.032]	−0.039*	[−0.084, 0.005]	−0.020	[−0.122, 0.081]
Theory of planned behaviour (TPB) related variables								
Attitude	0.051***	[0.022, 0.079]	0.0823**	[0.008, 0.156]	0.007	[−0.032, 0.047]	−0.099*	[−0.205, 0.007]
Perceived behaviour control	0.023***	[0.010, 0.037]	0.00984	[-0.027, 0.046]	0.011	[−0.013, 0.034]	−0.030	[−0.086, 0.026]
Self-efficacy	−0.145***	[−0.184, −0.107]	−0.0543	[−0.134, 0.025]	−0.098**	[−0.178, −0.018]	−0.0017	[−0.123, 0.119]
Risk perception								
Pandemic concern	0.121***	[0.059, 0.182]	0.109	[−0.132, 0.350]	−0.002	[−0.178, −0.018]	0.051	[−0.226, 0.327]
Pandemic severity	0.022**	[0.001, 0.043]	0.0462	[−0.019, 0.112]	−0.005	[−0.039, 0.030]	0.065	[−0.100, 0.230]
Vaccine safety	0.097***	[0.025, 0.169]	−0.0120	[−0.169, 0.145]	0.122*	[−0.039, 0.030]	−0.093	[−0.417, 0.231]
Vaccine effectiveness	0.017	[−0.041, 0.075]	0.140	[−0.031, 0.312]	0.052	[−0.053, 0.156]	0.018	[−0.145, 0.180]
Vaccine necessity	0.358***	[0.259, 0.458]	0.314**	[0.035, 0.593]	0.171*	[−0.011, 0.353]	0.611***	[0.186, 1.037]
Behavioural interventions								
Positive incentives	0.021*	[−0.001, 0.042]	−0.0378	[-0.095, 0.019]	−0.022	[−0.011, 0.353]	0.064	[−0.016, 0.144]
Negative pressure	−0.006	[−0.026, 0.015]	−0.00173	[-0.062, 0.058]	0.046**	[0.010, 0.082]	−0.057	[−0.141, 0.027]
Information prompt	0.030*	[−0.000, 0.060]	−0.0418	[-0.111, 0.028]	0.055*	[−0.007, 0.117]	−0.000	[−0.089, 0.088]
Vaccination prompt	0.064***	[0.036, 0.092]	0.159***	[0.080, 0.238]	0.055*	[−0.007, 0.116]	0.090	[−0.022, 0.202]
Demographic characteristic								
Gender	0.048*	[−0.006, 0.102]	−0.0573	[−0.207, 0.092]	0.024	[−0.071, 0.119]	−0.268**	[−0.512, −0.024]
Age	0.000	[−0.002, 0.002]	−0.003	[−0.039, 0.033]	−0.025***	[−0.071, 0.119]	−0.001	[−0.028, 0.025]
Education	−0.029**	[−0.055, −0.002]	0.021	[−0.052, 0.094]	0.014	[−0.034, 0.062]	0.002	[−0.093, 0.098]
Political statue	−0.012	[−0.028, 0.004]	0.0359*	[−0.007, 0.078]	−0.014	[−0.034, 0.062]	0.052	[−0.013, 0.116]
Income	0.008	[−0.011, 0.027]	−0.009	[-0.089, 0.071]	0.005	[-0.029, 0.040]	−0.049	[−0.148, 0.051]
Contraindication	−0.064***	[−0.093, −0.034]	−0.092***	[−0.154, −0.029]	−0.058**	[-0.029, 0.040]	0.044	[−0.030, 0.118]
Observations	6,525		879		1,266		191	
Pseudo R-squared	0.103		0.120		0.126		0.208	

Data collected in C city, China, during the national elderly and booster vaccination campaign (see Table 1 for details).

Standard errors in parentheses *p < 0.1, **p < 0.05, ***p < 0.01.

**TABLE 6:** Average marginal effects from the probit model of vaccination behaviour among older adults (n = 8,525). (Beijing, China, 2022).

Vaccination Behaviour								
Variables	≥18		61–65		66–75		>75	
	AME	95% CI	AME	95% CI	AME	95% CI	AME	95% CI
Vaccine hesitancy	0.018***	[0.015, 0.021]	0.025***	[0.014, 0.036]	0.027***	[0.019, 0.035]	0.051***	[0.016, 0.086]
Health belief model (HBM) related variables								
Perceived susceptibility	−0.003*	[-0.006, 0.000]	−0.010*	[−0.022, 0.001]	0.002	[−0.004, 0.009]	−0.012	[−0.031, 0.007]
Perceived severity	0.008***	[0.005, 0.011]	0.018***	[0.007, 0.029]	−0.001	[−0.007, 0.006]	0.033***	[0.009, 0.058]
Perceived benefits	0.001	[-0.003, 0.004]	0.002	[−0.012, 0.015]	0.007	[−0.001, 0.015]	0.014	[−0.012, 0.039]
Perceived barriers	0.000	[-0.005, 0.005]	−0.002	[−0.018, 0.014]	0.001	[−0.010, 0.012]	0.015	[−0.013, 0.042]
Theory of planned behaviour (TPB) related variables								
Attitude	0.005**	[0.001, 0.010]	0.023**	[0.005, 0.041]	0.003	[−0.006, 0.012]	0.016	[−0.008, 0.040]
Perceived behaviour control	0.005***	[0.002, 0.008]	0.018***	[0.007, 0.029]	0.007**	[0.000, 0.013]	0.008	[−0.013, 0.028]
Self-efficacy	0.004	[-0.001, 0.010]	−0.016	[−0.034, 0.003]	0.002	[-0.009, 0.012]	−0.019	[−0.046, 0.008]
Risk perception								
Pandemic concern	−0.008*	[−0.017, 0.001]	−0.029	[-0.064, 0.012]	−0.000	[−0.029, 0.029]	0	--
Pandemic severity	−0.002	[−0.006, 0.002]	0.003	[-0.008, 0.014]	−0.001	[−0.009, 0.007]	−0.010	[−0.045, 0.025]
Vaccine safety	0.00597*	[−0.001, 0.013]	0.018*	[-0.002, 0.037]	−0.007	[−0.024, 0.009]	−0.033	[−0.133, 0.068]
Vaccine effectiveness	−0.000	[−0.007, 0.006]	0.011	[-0.009, 0.032]	0.008	[−0.005, 0.021]	0.001	[−0.043, 0.044]
Vaccine necessity	−0.010**	[−0.019, −0.000]	−0.042**	[-0.075, −0.009]	−0.003	[−0.024, 0.017]	−0.044	[−0.127, 0.038]
Behavioural interventions								
Positive incentives	−0.003	[−0.007, 0.001]	−0.005	[−0.017, 0.008]	0.002	[−0.005, 0.009]	−0.012	[−0.043, 0.019]
Negative pressure	−0.002	[−0.006, 0.001]	−0.009	[−0.022, 0.005]	−0.006	[−0.015, 0.004]	−0.016	[−0.040, 0.008]
Information prompt	−0.000	[−0.005, 0.004]	−0.001	[−0.015, 0.013]	0.001	[−0.010, 0.011]	−0.016	[−0.040, 0.008]
Vaccination prompt	−0.001	[−0.006, 0.004]	0.005	[−0.008, 0.017]	−0.008	[−0.020, 0.005]	−0.012	[−0.042, 0.018]
Demographic characteristic								
Gender	−0.013***	[−0.023, −0.003]	−0.048**	[−0.089, −0.007]	−0.009	[−0.029, 0.012]	0.053	[−0.025, 0.131]
Age	−0.000**	[−0.001, −0.001]	−0.014***	[−0.024, −0.004]	−0.002	[−0.006, 0.001]	−0.005	[−0.015, 0.005]
Education	0.004**	[0.000, 0.009]	−0.006	[−0.018, 0.017]	0.011**	[0.001, 0.020]	0.023**	[0.000, 0.047]
Political statue	−0.001	[−0.004, 0.002]	−0.002	[−0.011, 0.007]	0.004	[−0.003, 0.010]	−0.011	[0.000, 0.047]
Income	0.001	[−0.003, 0.004]	0.000	[−0.017, 0.017]	−0.004	[−0.010,0.005]	0.020*	[−0.003, 0.043]
Contraindication	−0.024***	[−0.028, −0.020]	−0.037***	[−0.051, −0.024]	−0.022***	[-0.031, −0.013]	−0.047***	[−0.075, −0.018]
Observations	6,525		879		1,266		191	
Pseudo R-squared	0.103		0.120		0.126		0.208	

Data collected in C city, China, during the national elderly and booster vaccination campaign (see Table 1 for details).

Standard errors in parentheses *p < 0.1, **p < 0.05, ***p < 0.01.

In the Vaccine Hesitancy survey, several variables show statistically significant associations with vaccine hesitancy in the full sample. Specifically, Attitude (β = 0.045, p < 0.1, AME = 0.051, 95% CI: 0.022–0.079), Pandemic Concern (β = 0.105, p < 0.01, AME = 0.121, 95% CI: 0.059–0.182), Pandemic Severity (β = 0.059, p < 0.01, AME = 0.022, 95% CI: 0.001–0.043), Vaccine Safety (β = 0.126, p < 0.01, AME = 0.097, 95% CI: 0.025–0.169), Vaccine Necessity (β = 0.341, p < 0.01, AME = 0.358, 95% CI: 0.259–0.458), Information Prompt (β = 0.062, p < 0.01, AME = 0.030, 95% CI: -0.000–0.060), and Vaccination Prompt (β = 0.173, p < 0.01, AME = 0.064, 95% CI: 0.036–0.092) are positively associated with vaccine hesitancy. In contrast, Perceived Barriers (β = −0.155, p < 0.01, AME = −0.068, 95% CI: -0.096 to −0.040) and Self-Efficacy (β = −0.183, p < 0.01, AME = −0.184, 95% CI: -0.184 to −0.107) are negatively associated with vaccine hesitancy. Compared to the full sample, the influence of these variables on vaccine hesitancy is somewhat reduced in the older adult subgroups. However, Attitude, Vaccine Necessity, Positive Incentives, Negative Peer Pressure, and Vaccination Prompt exhibit higher significance and AMEs in the older adult groups, particularly in the behavioral intervention group, which shows better predictive value for older adults’ vaccine hesitancy.

In the analysis of Vaccination Behavior, Vaccine Hesitancy remains significantly positively associated with vaccination behavior across all age groups (p < 0.01). Furthermore, Perceived Severity (β = 0.063, p < 0.01, AME = 0.008, 95% CI: 0.005–0.011), Attitude (β = 0.123, p < 0.01, AME = 0.005, 95% CI: 0.001–0.010), and Perceived Behavioral Control (β = 0.029, p < 0.01, AME = 0.005, 95% CI: 0.002–0.008) are significantly associated with vaccination behavior in the full sample. Other variables such as Perceived Benefits (β = −0.036, p < 0.05, AME = 0.001, 95% CI: -0.003–0.004), Perceived Barriers (β = −0.054, p < 0.05, AME = 0.000, 95% CI: -0.005–0.005), Self-Efficacy (β = 0.103, p < 0.01, AME = 0.004, 95% CI: −0.001–0.010), Positive Incentives (β = −0.051, p < 0.01, AME = −0.003, 95% CI: -0.007–0.010), and Vaccination Prompt (β = 0.070, p < 0.01, AME = −0.001, 95% CI: -0.006–0.004) show inconsistent statistical significance, suggesting that their predictive relationships with vaccination behavior are not robust.

Among the control variables, some noteworthy findings emerge. Overall, control variables have a more significant impact on vaccination behavior than on vaccine hesitancy. Notably, contraindications are the most influential factor, significantly reducing both vaccine hesitancy and vaccination behavior across almost all age groups, except for individuals aged 75 and older. Additionally, younger age, male gender, and lower education levels are associated with higher vaccine hesitancy and more proactive attitudes toward vaccination. However, income level does not show a significant association with vaccine hesitancy or vaccination behavior (p > 0.1). In summary, psychological variables demonstrate sufficient explanatory power for vaccine hesitancy and vaccination behavior across the entire population, while behavioral interventions exhibit better predictive value for older adults.

## Discussion

The World Health Organization (WHO) identified vaccine hesitancy as one of the top ten global health threats in 2019. Consequently, governments must focus on determining the causes of vaccine hesitancy and employing effective solutions to address this issue [65]. China’s extensive immunization program in response to the pandemic has resulted in high vaccination rates. In comparison, older adults have received less attention, posing obstacles for government recognition and promotion of vaccination in this age group. This study addresses this gap by identifying predictors of vaccine hesitancy and vaccination behavior across age groups, with a focus on adults aged 60 and above.

Within our sample, individuals in China demonstrated a strong willingness to receive vaccination, and with a small portion either absolutely unwilling or possibly unwilling to receive the pandemic vaccine. It is important to note that the timing and location of data collection can lead to varying conclusions regarding vaccine hesitancy prevalence [66], as attitudes toward vaccination have evolved throughout different stages of the pandemic and in response to various policies. While the independent variables selected for this study have been widely recognized as influential in prior research, our findings revealed some distinct characteristics among the elderly compared to the younger adult sample. In addition to age, gender and education level were also associated with hesitancy and uptake rates, with males and individuals with lower education levels showing relatively lower hesitancy and higher vaccination behavior. These gender differences align with previous studies [67–69]. Females were more likely to be hesitant due to concerns about vaccine efficacy and a fear of injections [11, 70]. Regarding the relationship between education level and hesitancy, our results diverged from previous studies, which generally consider lack of education as a barrier to vaccination during the pandemic [71]. In our study, a higher education level was associated with a significant decrease in vaccination willingness, and the reason for this unexpected finding requires further exploration.

Our study found that factors affecting vaccine hesitancy and vaccination behavior in older include perceived severity, perceived barriers, attitude, self-efficacy, vaccination prompts, and contraindications. Probit regression analysis showed that in older adults, vaccine hesitancy was significantly associated with Perceived Benefits, Perceived Barriers, Attitude, Self-Efficacy, Vaccine Safety, Vaccine Necessity, Positive Incentives, Negative Pressure, Information Prompt And Vaccination Prompt. Regarding Vaccination Behavior, Perceived Severity, Perceived Benefits, Perceived Barriers, Attitude, Perceived Behaviour Control, Self-Efficacy and Positive Incentives were stronger motivators for older adults than for the overall sample aged 18 and above to promote vaccination. Previous research has noted that misinformation about vaccine side effects, such as heart attacks and severe allergies, has negatively affected vaccination intentions among older adults [72]. However, our study indicates that older adults’ attitudes toward vaccines are rational and realistic. They are more concerned with the severity of the pandemic and the necessity of vaccination than with vaccine safety and efficacy. Additionally, older adults are particularly concerned about vaccination convenience. Factors such as improving vaccination accessibility, reducing vaccination barriers, providing health information, and delivering vaccination reminders can significantly enhance vaccine acceptance and actual vaccination behavior among older adults. The significant role of vaccination accessibility and reminder systems suggests that structural and logistical factors play a key part in shaping vaccine acceptance among older adults. Poor health status and limited mobility may make it difficult for this population to reach vaccination sites, particularly if they rely on public transportation. Therefore, interventions that reduce physical and cognitive barriers—such as establishing community-based clinics, offering transportation support, and implementing personalized vaccination reminders—could enhance both perceived convenience and actual uptake. Moreover, the positive association between vaccination prompts and willingness highlights the importance of proactive communication. However, as such information is increasingly disseminated through digital platforms, older adults with limited digital literacy or internet access may be disadvantaged. Future efforts should ensure equitable access to vaccine information through multimodal channels, including traditional media and community outreach, to bridge the digital divide.

This study has several limitations. First, data were collected during China’s rollout of primary vaccination for adults aged 60 and older and booster doses for those aged 18–60, a period when vaccines had already been available for some time. As such, the findings may not generalize to earlier or later stages of the vaccination campaign or to other vaccine types and public health contexts. Second, due to the cross-sectional design, temporal precedence cannot be established, and causal inferences are not warranted. While our analysis identifies statistically significant associations between psychosocial factors and both vaccine hesitancy and vaccination behavior, longitudinal or experimental studies are needed to examine causal pathways. Third, the sample is not nationally representative, with a substantial proportion of respondents recruited from C city—a metropolitan area with high levels of education, healthcare access, and public health infrastructure. Consequently, the observed levels of vaccine hesitancy and the strength of associations may not be generalizable to populations in rural or less developed regions. Nevertheless, the inclusion of participants from 27 additional provinces enhances geographic diversity compared to single-site studies. Finally, while we examine both vaccine hesitancy and vaccination behavior, the precise mechanisms through which hesitancy translates into behavioral outcomes warrant further investigation using mediation or path analysis in larger, longitudinal datasets.

### Conclusion

This study identifies key cognitive, behavioral, and structural determinants of vaccine hesitancy and uptake during the pandemic among older adults in China. Probit regression analyses reveal that vaccine hesitancy is significantly associated with Perceived Susceptibility, Perceived Barriers, Attitude, Vaccine Necessity, and Vaccination Prompt. In contrast, actual vaccination behavior is more strongly predicted by Vaccine Hesitancy, Perceived Benefits, Perceived Behavioral Control, and Perceived Severity.

These findings underscore the importance of implementing targeted, multi-component interventions that simultaneously address informational gaps, attitudinal barriers, and practical access challenges. Effective strategies should incorporate tailored risk communication delivered through trusted interpersonal networks and traditional media, enhanced service accessibility via mobile clinics and community-based vaccination programs, and active engagement of family members, who often play a decisive role in older adults’ health-related decisions.

Future research should employ longitudinal designs to examine the dynamic evolution of vaccine hesitancy and vaccination behavior over time. To improve generalizability, studies should expand sampling to underrepresented rural and underserved populations, ideally through stratified random sampling across urban, suburban, and rural settings. Furthermore, rigorous evaluation—using randomized controlled trials or quasi-experimental designs—is needed to assess the effectiveness of behavioral nudges and policy-level interventions in reducing vaccine hesitancy among older adults and other vulnerable groups. Mediation analyses can also help clarify the pathways linking psychological determinants to actual vaccination behavior.
